# Left apicoposterior segmentectomy for lung cancer with displaced segmental bronchus: a case report

**DOI:** 10.1186/s13019-020-01328-3

**Published:** 2020-09-29

**Authors:** Masahiro Yanagiya, Hirokazu Yamaguchi, Noriko Hiyama, Jun Matsumoto

**Affiliations:** 1 Department of General Thoracic Surgery, NTT Medical Center Tokyo, 5-9-22 Higashi-Gotanda, Shinagawa-ku, Tokyo, 141-8625 Japan; 2 Department of Thoracic Surgery, The University of Tokyo Graduate School of Medicine, Tokyo, Japan

**Keywords:** Thoracic surgery, Lung cancer, Segmentectomy, Bronchial anomaly

## Abstract

**Background:**

Pulmonary segmentectomy can be challenging when thoracic surgeons encounter anatomical anomalies. A displaced left apicoposterior bronchus is a rare bronchial anomaly that makes lung anatomical resection challenging. We herein present a case of successful left apicoposterior segmentectomy for lung cancer in a patient with an anomalous segmental bronchus.

**Case presentation:**

A 70-year-old man was clinically diagnosed with early-stage lung cancer for which segmentectomy was indicated. A preoperative image revealed a displaced left apicoposterior bronchus that branched behind the left main pulmonary artery. With the aid of three-dimensional reconstruction imaging and systemic indocyanine green injection, we successfully performed left apicoposterior segmentectomy under complete video-assisted thoracic surgery. The pathological diagnosis was adenocarcinoma. The patient was alive without recurrence 8 months after segmentectomy.

**Conclusion:**

Preoperative three-dimensional imaging and systemic indocyanine green injection enabled us to successfully conduct challenging segmentectomy in a patient with an anomalous bronchus.

## Background

Although the optimal surgical treatment for lung cancer has long been lobectomy, segmentectomy may become a standard treatment for early-stage lung cancer [1]. Previous studies have shown that segmentectomy is oncologically comparable with lobectomy for early-stage lung cancer [2]. Segmentectomy also has the advantage of preservation of lung function [2, 3].

Segmentectomy requires a higher degree of skill than lobectomy for thoracic surgeons [1]. Moreover, segmentectomy is often challenging when thoracic surgeons encounter anatomical anomalies during surgery. A displaced left apicoposterior bronchus (B^1 + 2^) is a bronchial anomaly that thoracic surgeons sometimes encounter [4]. Although previous reports have described lobectomy for lung cancer with a displaced left B^1 + 2^, few reports have described segmentectomy for an anomalous bronchial branch [5–7]. We herein report a case of successful left apicoposterior segmentectomy for lung cancer in a patient with a displaced segmental bronchus using video-assisted thoracic surgery (VATS) with the aid of recently developed advanced techniques.

## Case presentation

**Fig. 1:**
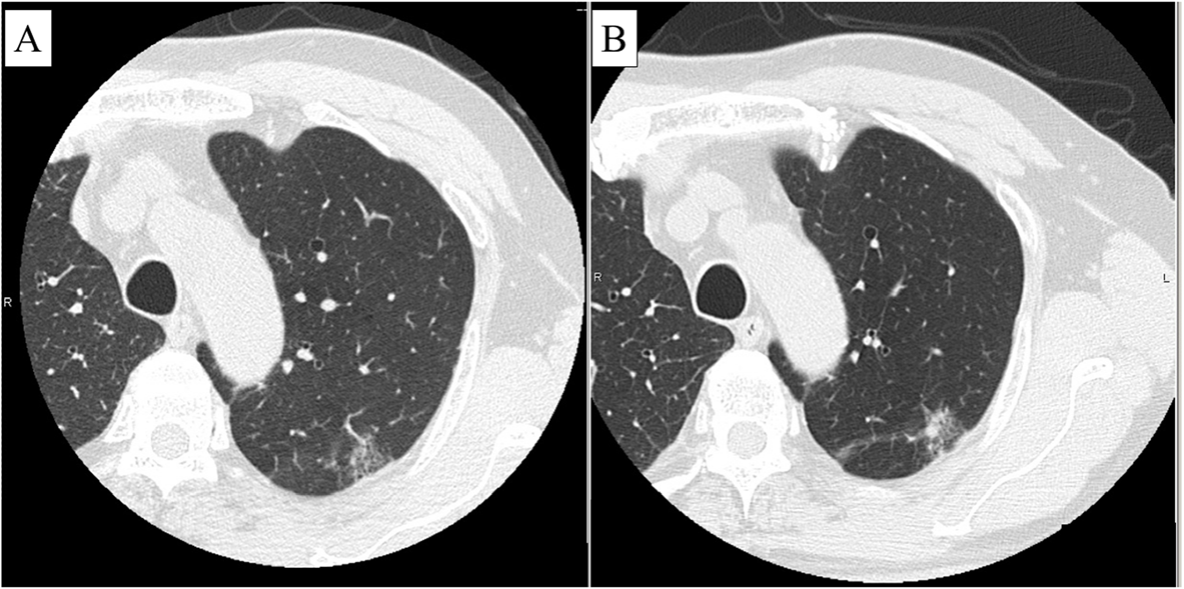
Preoperative chest computed tomography images. **a** Initially, chest computed tomography showed a pure ground-glass nodule with a possibility of benignity. **b** Six months later, chest computed tomography revealed a part-solid ground-glass nodule containing a solid component that was highly suspicious for malignancy

A 70-year-old man with no symptoms and a history of diabetes mellitus and subsequent chronic kidney disease was referred to our hospital because an abnormal lung nodule had been detected by chest computed tomography (CT). Initially, the CT image revealed a pure ground-glass nodule that was thought to be benign (Fig. 1a). During 6 months of close follow-up, the nodule gradually developed a solid component. CT finally showed a part-solid ground-glass nodule measuring 22 mm (the solid component measured 8 mm) in the left apicoposterior segment (S^1 + 2^), which raised suspicion for malignancy (Fig. 1b). 18F-fluorodeoxyglucose positron emission tomography (FDG-PET) showed hypometabolic activity (maximum standardized uptake value, 1.4). Distant metastases were not detected by whole-body CT or FDG-PET. The patient was referred to our department for surgical treatment.

**Fig. 2:**
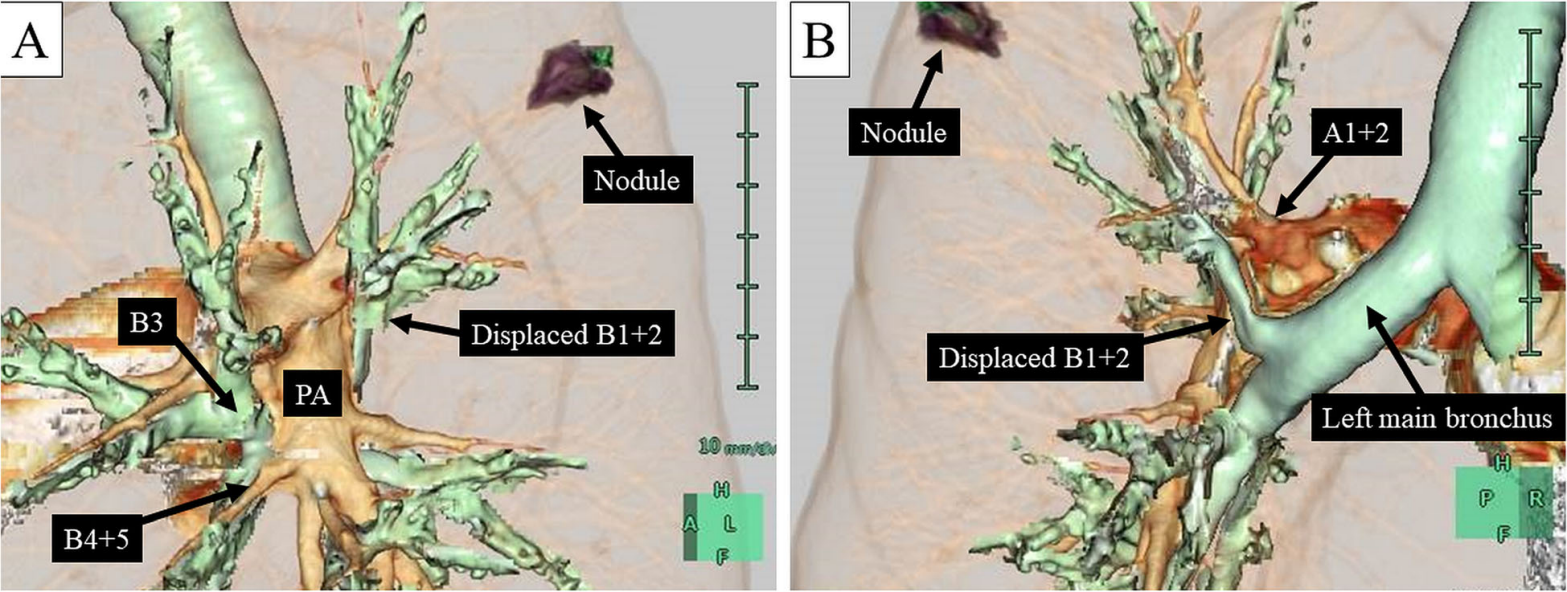
Preoperative three-dimensional chest computed tomography images. **a** Preoperative three-dimensional computed tomography reconstruction imaging revealed a displaced apicoposterior bronchus (B^1 + 2^) branching behind the main pulmonary artery. The nodule was located in the left apicoposterior segment (A^1 + 2^). **b** A branch of the pulmonary artery of the left A^1 + 2^ branched from the main pulmonary artery along the head side of the displaced B^1 + 2^. PA: pulmonary artery

The preoperative CT scan showed a displaced anomalous B^1 + 2^ branching from the left main bronchus behind the left main pulmonary artery (Fig. 2a, b). The patient was suspected to have early-stage lung cancer (cT1aN0M0-IA1) located in S^1 + 2^ with a left displaced anomalous B^1 + 2^.

**Fig. 3:**
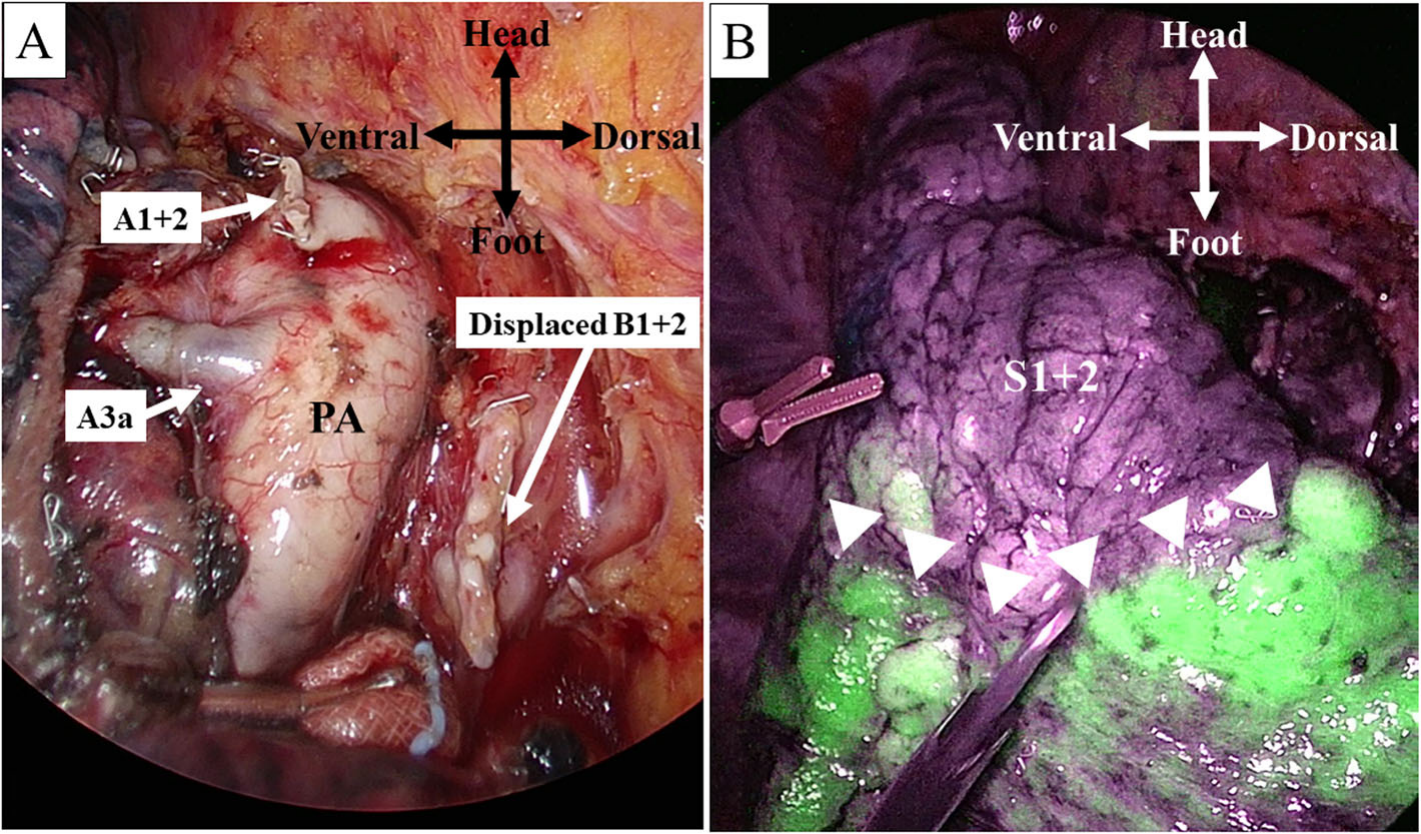
Intraoperative view of surgical field. **a** Photograph after dissection of displaced apicoposterior bronchus (B^1 + 2^) and apicoposterior segement (A^1 + 2^). **b** Delineation of intersegmental plane by systemic indocyanine green injection under near-infrared imaging. The white arrows suggest the intersegmental plane. PA: pulmonary artery

Considering the patient’s comorbidity, we decided to perform left S^1 + 2^ segmentectomy. The surgery was conducted under four-port VATS. The displaced B^1 + 2^ was initially accessed by dissecting along the posterior side of the mediastinal pleura. We identified the displaced B^1 + 2^ and subsequently detected A^1 + 2^ branching along the displaced B^1 + 2^ from the left main pulmonary artery. After dissecting the hilar lymph nodes, the displaced B^1 + 2^ and A^1 + 2^ were exposed and cut respectively with a mechanical stapler (Fig. 3a). Several lymph nodes were analyzed by intraoperative frozen section and found to be negative for metastasis. Indocyanine green (ICG) was administered intravenously. The intersegmental plane was identified under near-infrared thoracoscopy. The surface of the whole left lung except that of the target segment turned green (Fig. 3b). In addition to the intersegmental plane, we identified the actual location of the tumor with the aid of palpation thoracoscopically. Following the intersegmental plane suggested by systemic ICG injection and after confirming the tumor location, we completed left S^1 + 2^ segmentectomy with the use of mechanical staplers. After obtaining the specimen, we reconfirmed intraoperatively that the surgical margin was tumor-free. The operation time was 130 min, and the blood loss was minimal. The postoperative course was uneventful, and the patient was discharged 4 days after surgery.

The pathological diagnosis was invasive adenocarcinoma. The dimension of tumor invasion was 16 mm. The surgical margin was negative and all lymph nodes were negative for metastases. The pathological stage was p-T1bN0M0. At the time of this writing (8 months postoperatively), the patient was alive without recurrence.

## Discussion

We have herein described the successful performance of left S^1 + 2^ segmentectomy for lung cancer with a displaced B^1 + 2^. As advancements in CT scanning continue to facilitate detection of many lung cancers indicated for segmentectomy, thoracic surgeons will increasingly encounter segmental bronchial anomalies. Thoracic surgeons should have a detailed knowledge of the anatomy of segmental bronchi, including their anomalies, to ensure appropriate performance of segmentectomy. The present case provides valuable information on how to manage a segmental bronchial anomaly during segmentectomy.

Preoperative three-dimensional CT reconstruction greatly contributes to establishment of the surgical strategy. In this case, we preoperatively determined that the anomalous B^1 + 2^ arose on the back of the left main pulmonary artery. This is why we initially accessed the displaced B^1 + 2^ from the posterior side. In some previous cases, the interlobar dissection approach resulted in accidental cutting of the displaced B^1 + 2^ involved with the lung parenchyma during anatomical resection for lung cancer [6, 7]. Preoperative three-dimensional reconstruction was useful in terms of understanding the anatomy in our case and thus helped us to avoid accidental cutting of the displaced B^1 + 2^.

Systemic ICG injection played an important role in this surgery. We easily identified the intersegmental plane with the aid of intraoperative ICG injection (Fig. 3b). Previous studies have demonstrated the efficacy of systemic ICG injection [8, 9]. Our case suggests that systemic ICG can be effective even in patients with a segmental bronchial anomaly. Systemic ICG injection does not require inflation to identify the segmental plane and creates more surgical space, thus facilitating complete VATS surgery [10]. Although a previous case report described segmentectomy for lung cancer with an anomalous segmental bronchus via open thoracotomy [6], we performed this challenging surgery via complete VATS with the assistance of systemic ICG injection.

## Conclusion

We performed successful left S^1 + 2^ segmentectomy for lung cancer in a patient with an anomalous segmental bronchus via complete VATS. Preoperative three-dimensional imaging and systemic ICG injection led to the success of this segmentectomy.

## Data Availability

All data generated or analyzed are included in this article.

## Abbreviations

B^1 + 2^: Apicoposterior bronchus
VATS: Video-assisted thoracic surgery
CT: Computed tomography
S^1 + 2^: Apicoposterior segment
FDG-PET: 18F-fluorodeoxyglucose positron emission tomography
ICG: Indocyanine green
